# LncRNA‐miRNA network analysis across the Th17 cell line reveals biomarker potency of lncRNA NEAT1 and KCNQ1OT1 in multiple sclerosis

**DOI:** 10.1111/jcmm.17256

**Published:** 2022-03-10

**Authors:** Elham Karimi, Hanieh Azari, Ahmad Tahmasebi, Amin Reza Nikpoor, Ahmad Agha Negahi, Nima Sanadgol, Mohammad Shekari, Pegah Mousavi

**Affiliations:** ^1^ Department of Medical Genetics Faculty of Medicine Hormozgan University of Medical Sciences Bandar Abbas Iran; ^2^ Institute of Biotechnology Shiraz University Shiraz Iran; ^3^ Sciences Research Center for Molecular Medicine Hormozgan University of Medical Hormozgan Iran; ^4^ Department of Internal Medicine Faculty of Medicine Hormozgan University of Medical Sciences Bandar Abbas Iran; ^5^ Institute of Neuroanatomy RWTH University Hospital Aachen Aachen Germany

**Keywords:** bioinformatics, differentiation, lncRNAs, multiple sclerosis, Th17/Treg imbalances

## Abstract

Differentiation of CD^4+^ T cells into Th17 cells is an important factor in the onset and progression of multiple sclerosis (MS) and Th17/Treg imbalance. Little is known about the role of lncRNAs in the differentiation of CD^4+^ cells from Th17 cells. This study aimed to analyse the lncRNA‐miRNAs network involved in MS disease and its role in the differentiation of Th17 cells. The lncRNAs in Th17 differentiation were obtained from GSE66261 using the GEO datasets. Differential expression of lncRNAs in Th17 primary cells compared to Th17 effector cells was investigated by RNA‐seq analysis. Next, the most highlighted lncRNAs in autoimmune diseases were downloaded from the lncRNAs disease database, and the most critical miRNA was extracted by literature search. Then, the lncRNA‐miRNA interaction was achieved by the Starbase database, and the ceRNA network was designed by Cytoscape. Finally, using the CytoHubba application, two hub lncRNAs with the most interactions with miRNAs were identified by the MCODE plug‐in. The expression level of genes was measured by qPCR, and the plasma level of cytokines was analysed by ELISA kits. The results showed an increase in the expression of NEAT1, KCNQ1OT1 and RORC and a decrease in the expression of FOXP3. In plasma, an upregulation of IL17 and a downregulation of TGFB inflammatory cytokines were detected. The dysregulated expression of these genes could be attributed to relapsing‐remitting MS (RR‐MS) patients and help us understand MS pathogenesis better.

## 1 INTRODUCTION

Multiple sclerosis (MS) is a progressive autoimmune disease characterized by inflammation of the central nervous system. Genetic and environmental factors determine individual susceptibility to MS as a multifactorial disease.^1^ CD^4+^ cells may play an essential role in the development of neuroinflammation. Although the exact mechanism of MS pathogenesis is not well understood, T cells appear to have a crucial function in this process. Studies have shown that in autoimmune diseases, especially MS, there is an imbalance between Th17 and Treg cells.^2^ Th17 cells increase inflammatory responses by releasing other inflammatory factors and attracting neutrophils. In addition, Th17 cells secrete IL‐17, which has a strong pro‐inflammatory effect. Conversely, Treg cells are primarily involved in regulating the immune response and the activity of other immune cells, thus limiting the immune response involving T cells to a safe range to prevent damage to the body. Treg and Th17 cells, which are antagonistic to each other, are in balance under normal conditions.^3^ However, recent evidence suggests that an imbalance between a Th17 and Treg plays a crucial role in the development of autoimmune diseases such as MS. Fork‐like transcription factor 3 (FOXP3) has been identified as an important molecular marker for Treg cells and as a major transcription factor influencing Treg cell function and growth. Downregulation of FOXP3 is associated with impaired differentiation of CD^4+^ T cells into Treg cells. The nuclear orphan receptor γt (RORC) is responsible for the differentiation of Th17 cells followed by RORC promotion. Therefore, regulation of FOXP3 and RORC expression would also affect the Th17/Treg balance.^4^ However, the biomolecule that could affect the expression of FOXP3 and RORC and thus disrupt the Th17/Treg balance at MS is still unknown. Long non‐coding RNA (lncRNA) is a class of non‐coding RNA that modulates biological activities such as altering histone and chromatin structure, altering splicing profiles, suppressing microRNA (miRNA) binding sites and regulating gene expression.^5^ The identification of aberrant expression of lncRNAs in the CNS and immune system, and its involvement in T cells and B cells differentiation and activation, make it one of the golden topics in the study of biomarker evaluation in neurological and autoimmune diseases, such as MS.^6^, ^7^ One of the most important lncRNAs is lincRNA‐Cox2, which regulates macrophages, and some lncRNAs expressed by T cells coincide with NRON, GAS5 and LincR‐Ccr2‐5'AS.^8^, ^9^ GAS5 are an essential suppressor of T‐cell proliferation and is associated with cell cycle suppression in response to stress or other environmental conditions; GAS5 also regulates glucocorticoid receptor expression.^10^ The upregulation of BACE1‐AS in Alzheimer's disease triggers an increase in BACE1 protein levels by binding and stabilizing BACE1 mRNA.^11^ Despite ample evidence for the involvement of lncRNAs in disease pathogenicity, especially in human autoimmunity, its role in the Th17 /Treg imbalance at MS remains controversial and should be investigated, which is essential for the development of targeted therapies. CD^4+^ T helper cells (Th), which are inflammatory and autoreactive that play an important role in the pathogenesis of MS.^12^ Recent studies have shown that lncRNAs are involved in regulating the function of CD^4+^ cells such as Th17/Treg. However, it is not yet known whether abnormal lncRNAs indirectly control FOXP3 and RORC levels during the development of MS, resulting in Th17/Treg imbalance.^13^ This study aimed to evaluate the association of lncRNAs with Th17/Treg imbalance in MS. Therefore, using a combination of bioinformatics and literature review methods, we gathered a list of genes differentially expressed in MS compared to controls. The lncRNAs that are likely to be dysregulated in this pathway were determined. Next, confirmation assays such as real‐time PCR (RT‐PCR) and ELISA were completed to confirm our claim.

## 2 MATERIAL AND METHODS

### 2.1 Screening of dysregulated lncRNAs in Th17 differentiation

The lncRNA expression profile in T helper 17‐cell differentiation associated with RNA‐seq (GSE66261) was taken from the GEO database (https://www.ncbi.nlm.nih.gov/geo/), which serves as a public repository for curated gene expression datasets and original series and platform records.^14^ We then used the lncRNA disease database (http://www.cuilab.cn/LncRNAdisease) to select the lncRNAs that were validated in autoimmune diseases.^15^ All microRNAs involved in Th17 differentiation in various autoimmune diseases were found using a literature review on Th17 differentiation and microRNA in MS, rheumatoid arthritis (RA), systemic lupus erythematosus (SLE), inflammatory bowel disease (IBD) and diabetes.^16^ Subsequently, the Starbase database (http://starbase.sysu.edu.cn/) was used to determine the interaction between lncRNA‐miRNAs involved in Th17 cell differentiation and autoimmune diseases.^17^ The lncRNA‐miRNA network was designed using Cytoscape. Finally, using the CytoHubba application, two lncRNA hubs that have to react most strongly with miRNAs in differentiation were identified by the MCODE method.^18^

### 2.2 Sample collection and peripheral blood mononuclear cell isolation

We selected RR‐MS patients according to McDonald's criteria who were taking immunosuppressive drugs such as methyl and interferon; pregnant women; people with a history of other autoimmune diseases; or people with cancer (benign or malignant tumours) were excluded from the study. Patients attending Abu Raihan Hospital in Bandar Abbas, Iran, participated in this study. From 25 RR‐MS patients and 25 healthy controls, 10 mL of blood was collected, and PBMC was extracted. PBMC was extracted using the Ficoll‐Hypaque density gradient centrifugation protocol. The blood was diluted 1:1 with PBS and then carefully placed in Falcon tubes containing 2 mL of Ficoll‐Hypaque solution. It was then centrifuged at 956 *g* for 20 minutes. After centrifugation, the solution contained three different phases, the supernatant was frozen at −80°C, the PBMC obtained was washed again with PBS at 680 g for 10 minutes, and then the RNA was extracted from the cell pellet.

### 2.3 Total RNA extraction and cDNA synthesis

Extraction of total RNA was performed using the triazole method according to the protocol of the RNX‐PLUS kit (Sinaclon). Quantification of extracted RNAs was evaluated using Nanodrop (Thermo Scientific™), and RNA quality was assessed by electrophoresis on 2% agarose gels. The cDNA synthesis kit (TAKARA) was used to convert RNA cDNA, using an equal volume of RNA for all samples.

### 2.4 Primer design and real‐time PCR

Primer design is the most important factor in qPCR; a variety of software has been used for primer design such as primer3, primer BLAST, gene runner and oligo analyser. Among the common genes as internal control, the β‐actin gene was selected for this study because this gene has relatively stable expression in peripheral blood mononucleosis cells and is also suitable for normalizing the expression of cytokine genes. The following steps were performed: 15 minutes at ‘95°C’, 40 cycles of PCR, 15 seconds of denaturation at ‘95°C’, 30 seconds of annealing and finally 30 minutes of elongation at ‘72°C’. Genes were amplified using the RealQ Plus Master Mix Green Denmark. The 2^−ΔΔCt^ method was used to calculate the expression of genes and lncRNAs. The primer sequences are listed in Table 1.

**TABLE 1:** Real‐time PCR primers are used

Gene names	F/R	Sequence
Β‐Actin	F	AGCCTTCCTTCCTGGGCATGG
Β‐Actin	R	AGCACTGTGTTGGCGTACAGGTC
FOXP3	F	CAGCACATTCCCAGAGTTCCTC
FOXP3	R	GCGTGTGAACCAGTGGTAGATC
RORC	F	TCCCGAGATGCTGTCAAGTTC
RORC	R	CACTGGTTCCTGTTGCTGC
NEAT1	F	CCAGTGTGAGTCCTAGCATTGC
NEAT1	R	CCTGGAAACAGAACATTGGAGAAC
KCNQ1OT1	F	TGCAGAAGACAGGACACTGG
KCNQOT1	R	CTTTGGTGGGAAAGGACAGA

### 2.5 Plasma extraction

Whole blood samples (2 mL) were collected in (blood collection tubes) ethylenediaminetetraacetic acid (EDTA) anticoagulation tubes. The tubes were immediately centrifuged (1000*g*/min for 10 minutes) at 4°C, and plasma was collected. Subsequently, all separated plasma samples were stored at −80°C in RNase‐free microcentrifuge tubes.

### 2.6 Enzyme‐linked immunosorbent assay

The presence of inflammatory cytokines such as TGF‐β and interleukin‐17 (IL‐17) in the plasma samples was determined by enzyme‐linked immunosorbent assay (ELISA), using the Karmania ELISA kit according to the manufacturer's instructions (Karmania Pars Gene). Briefly, 100 µL of serum was loaded on 96 well plates pre‐coated with IFN‐γ and IL‐4 capture antibodies. After washing, HRP‐streptavidin labelled detection antibodies were added. Finally, the TMB/peroxide (ELISA substrate) was added to the reaction, and the OD was recorded at 450 nm.

### 2.7 Statistical analysis

For the analysis of the real‐time PCR data, preliminary tests were performed using the CtΔΔ method. To select the type of statistical test for statistical analysis, the Shapiro‐Wilk test was first used to examine the normal distribution of the data. *p* values of ≤0.05 were considered as normal distribution and above that as abnormal distribution. The Mann‐Whitney U test was used to compare gene expression in the patient and control groups for abnormal data and Student's t‐test for normal data. Depending on the distribution of the data, data were defined as mean and standard deviation (SD), median and interquartile range (IQR), or number (per cent). The relationship between lncRNAs and genes expression was examined using Spearman's rank correlation to determine the degree of correlation between the variables. The efficiency of lncRNAs and genes in MS diagnosis was assessed using receiver operating characteristic curves (ROC) the area under the ROC curve (AUC) and the 95% confidence interval (CI). For the above analyses, we used SPSS 17.0 and GraphPad Prism 5.0. Statistical significance was described as a *p* value of less than 0.05.

## 3 RESULTS

### 3.1 Patient characteristics and their relation to genes expression in RR‐MS patients

**FIGURE 1:**
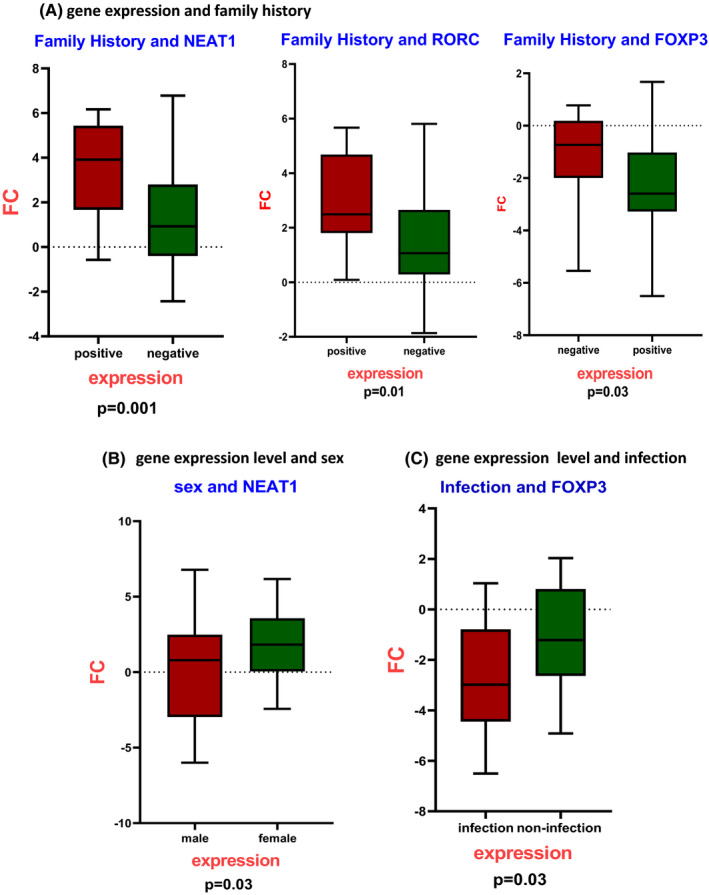
The relationship between deregulated genes and demographic data suggested the possible relations between deregulation genes and sex, viral infections and positive family history of autoimmune diseases. A, The significant relationship between NEAT1 (*p* < 0.001), RORC (*p* < 0.01) and FOXP3 (*p* < 0.03) expression and positive family history of autoimmune diseases. B, The upregulation of NEAT1 in women compared to men (*p* < 0.03). C, The significance of FOXP3 downregulation in MS patients who were affected by viral infections compared to MS patients with no infection history (*p* < 0.03)

The demographic data, sex, age, smoking, infections and family autoimmune history of 25 RR‐MS patients and 25 healthy controls are summarized in Table 2. Statistical analyses showed no significant differences between age and smoking in the sample group, but there was a significant association between the expression of NEAT1 (*p* < 0.001), RORC (*p* < 0.01) and FOXP3 (*p* < 0.03) and a positive family history of autoimmune diseases. In addition, downregulation of the FOXP3 gene was significant in RR‐MS patients recently affected by viral infections compared to MS patients with no history of infection (*p* < 0.03). The study shows that the expression level of the lncRNA NEAT1 is significantly increased in women compared to men (*p* < 0.03) (Figure 1).

**TABLE 2:** The demographic characteristic of individuals

Characteristics	Frequency (%)	Frequency (%)
	Case	Control
Sex		
Female	12 (48%)	12 (48%)
Male	13 (52%)	13 (52%)
Age		
≤30	15 (60%)	15 (60%)
˃30	10 (40%)	10 (40%)
Smoking		
Smoker	8 (32%)	—
Non‐smoker	17 (68%)	25 (100%)
Infection		
Yes	6 (24%)	—
No	19 (76%)	25 (100%)
Family history		
Positive	8 (32%)	4 (16%)
Negative	17 (68%)	21 (84%)

### 3.2 Identification of Th17‐specific lncRNAs and miRNAs differentially expressed

We first obtained the expression profile of Th17 cell differentiation RNA‐seq of GSE66261 from the GEO database using 51109 lncRNAs with GEO2R. Then, 81 lncRNAs specific for Th17 lineage were extracted by literature search and lncRNA disease database. The 320 microRNAs in autoimmune diseases and Th17 were archived by a literature search in MS, SLE, IBD, psoriasis, RA and diabetes. All previously discussed differentially expressed biomolecules were used to construct the dysregulated network.

### 3.3 Screening of DE‐lncRNAs in primary and effector Th17 cells

**FIGURE 2:**
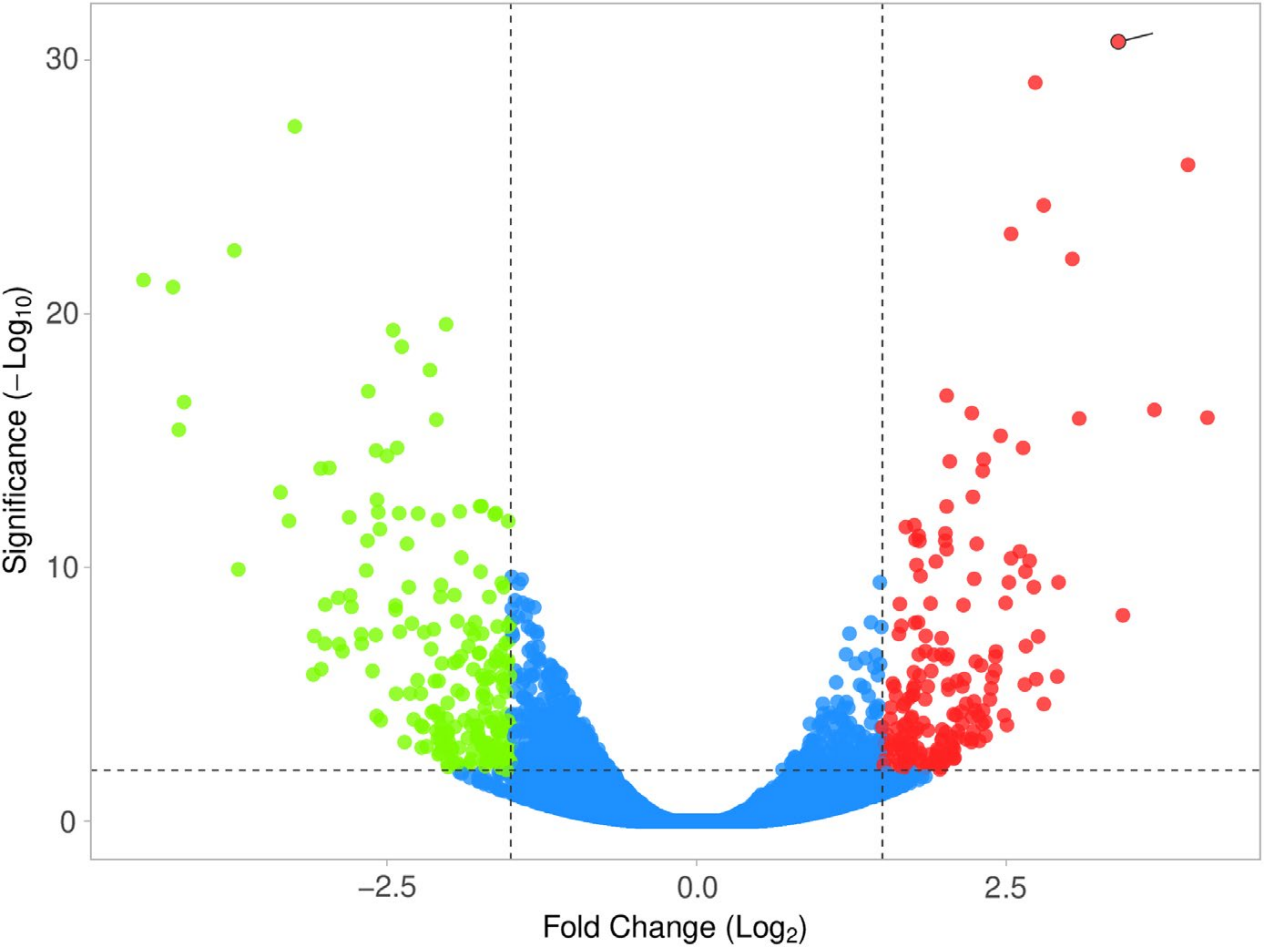
Volcano plot of differentially expressed lncRNAs. Upregulated and downregulated genes are represented by red and green dots, respectively. The blue dots represent no significant genes (at adjusted *p* < 0.05)

**FIGURE 3:**
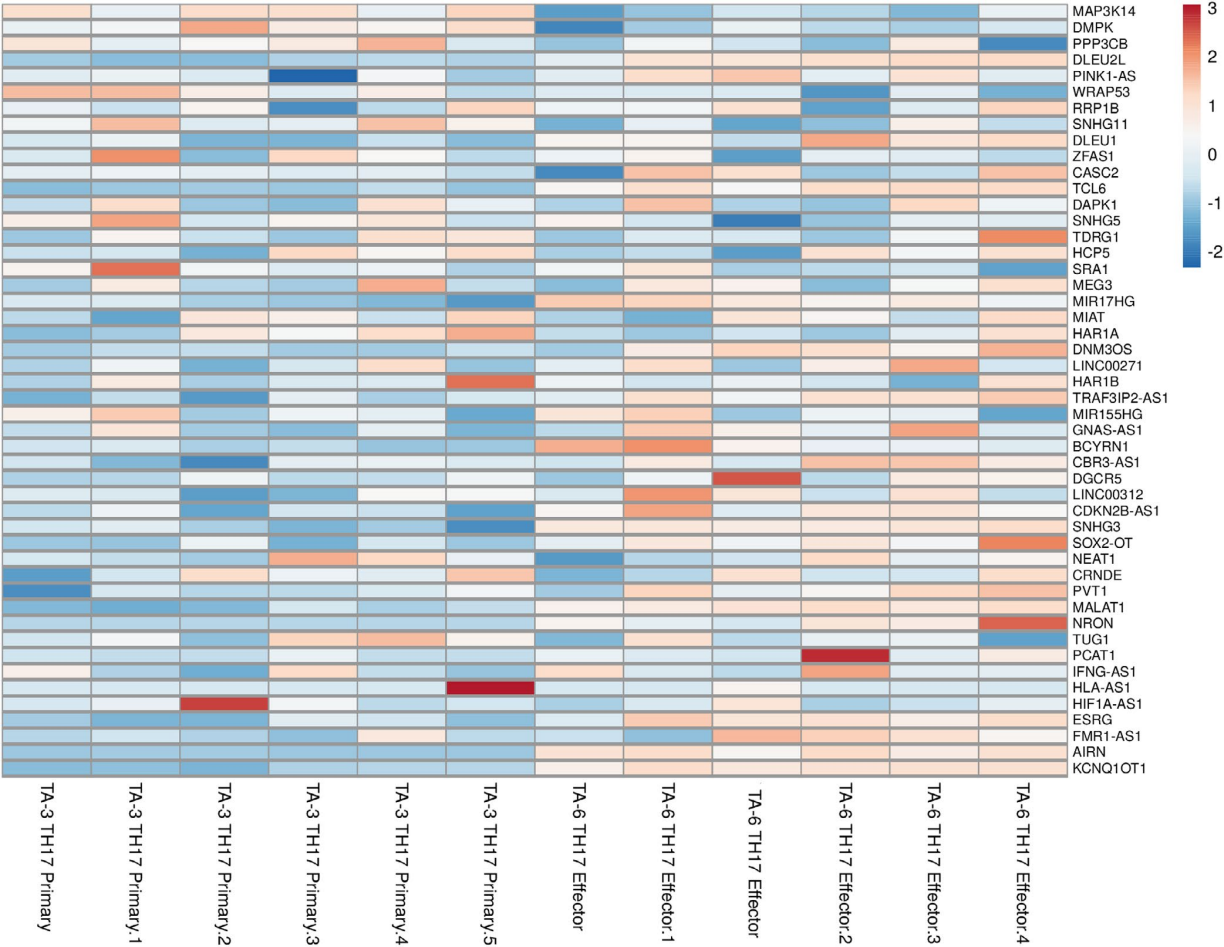
Heatmap of 48 DElncRNAs in different autoimmune diseases and Th17 cell linage. The rows in the heatmap represent the individual lncRNA IDs, and the columns represent the samples

To investigate the expression profile of lncRNAs in primary and effector Th17 cells, we retrieved the GSE66261 dataset from the NCBI Gene Expression Omnibus (GEO). Differential expression analysis between primary and effector Th17 cells was performed using DESeq2 (18). Genes with *p* adjusted <0.05 were considered significantly differentially expressed genes (DEGs). With a total of 51109 differentially expressed lncRNAs, we identified 48 DE lncRNAs between primary and effector Th17 cells and miRNA‐lncRNA constructs in autoimmune diseases (Figure 2). A heat map was also created to visualize the expression patterns of lncRNAs in different samples (Figure 3).

### 3.4 Analysis of DElnRNA‐DEmiRNA network demonstrates top‐five deregulated lncRNAs

**FIGURE 4:**
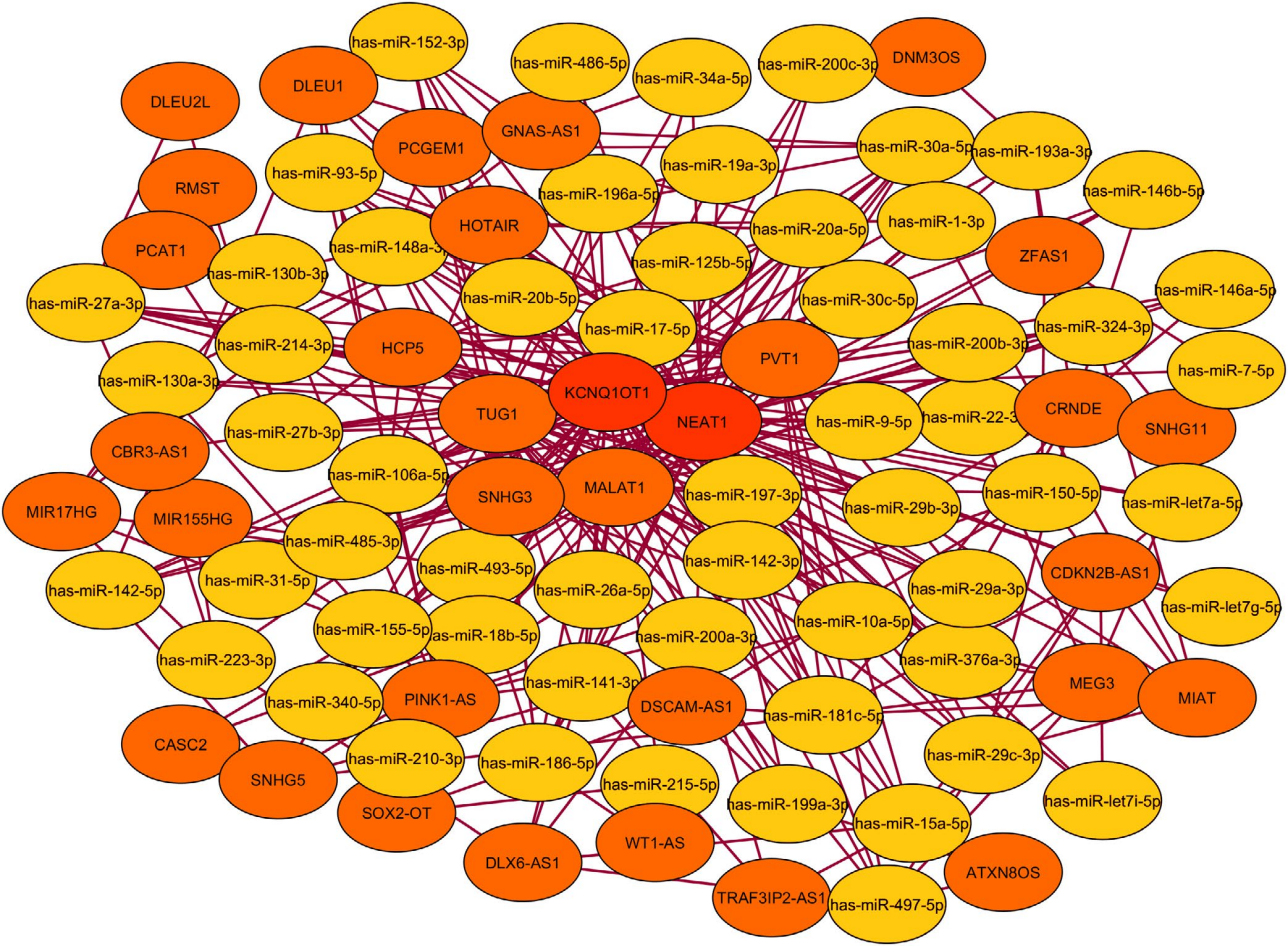
The lncRNA‐miRNA‐ceRNA network clarified the high interaction between DElncRNAs and DE miRNAs involved Th17 differentiation and autoimmune diseases. The yellow cycle is represented that miRNAs and LncRNAs are shown with the orange cycle. The most interactive lncRNA was marked in bold

The lncRNA‐miRNAs interaction was predicted by the Starbase database. Finally, the DElncRNA‐DEmiRNA network was created (Figure 4). The DElncRNA‐DEmiRNA network, which consists of 91 nodes, and 320 edges, was reconstructed and visualized using Cytoscape. The DEmiRNA and DElnRNA with high interaction levels were identified as hub nodes in the DElncRNA‐DEmiRNA regulatory network. The five highly interacting lncRNA included KCNQ1OT1, NEAT1, MALAT1, TUG1 and PVT1 (Table 3).

**TABLE 3:** Top 10 highly interacting ncRNAs in DElnRNA‐DEmiRNA network ranked by MCC method

Rank	Name	Score
1	KCNQ1OT1	54
2	NEAT1	53
3	MALAT1	26
4	TUG1	25
5	PVT1	19
6	HOTAIR	13
6	HCP5	13
8	SNHG3	11
9	PINK1‐AS	10
9	has‐miR‐181c‐5p	10

### 3.5 Upregulation of lncRNA NEAT1 and KCNQ1OT1 is strongly correlated with RR‐MS patients

NEAT1 and KCNQ1OT1 were detected by real‐time PCR in 25 relapsing‐remitting (RR) MS patients and 25 control subjects of the same age. The expression levels of NEAT1 were significantly higher in the PBMC of patients with RR‐MS compared to controls (*p* = 0.0002, FC = 1.78); the expression levels of KCNQ1OT1 were also significantly upregulated in RR‐MS patients (*p* < 0.0001, FC = 4.01). Surprisingly, there is a statistically significant positive correlation between the expression levels of these two lncRNAs (*p* < 0.0001, *R* = 0.52).

### 3.6 Altered expression of the FOXP3 and RORC as specific TFs in Th17/Treg imbalance is observed in RR‐MS patients

**FIGURE 5:**
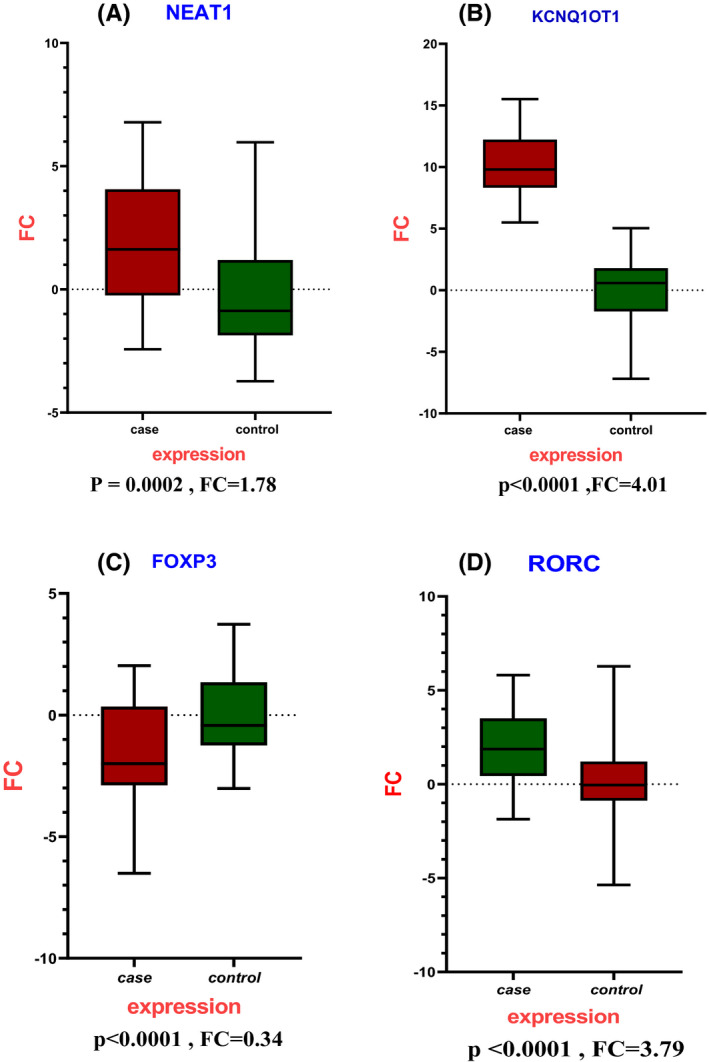
Expression results of lncRNAs and genes indicate a significant relationship between deregulation expression in MS. A, Increase expression of lncRNAs NEAT1 (*p* = .0002) and KCNQ1OT1 (*p* < .0001) in RR‐MS patients compared to healthy control. B, downregulation of FOXP3 (*p* < .0001) and upregulation of RORC (*p* < .0001) expression profile in RR‐MS patients compared to healthy control

**FIGURE 6:**
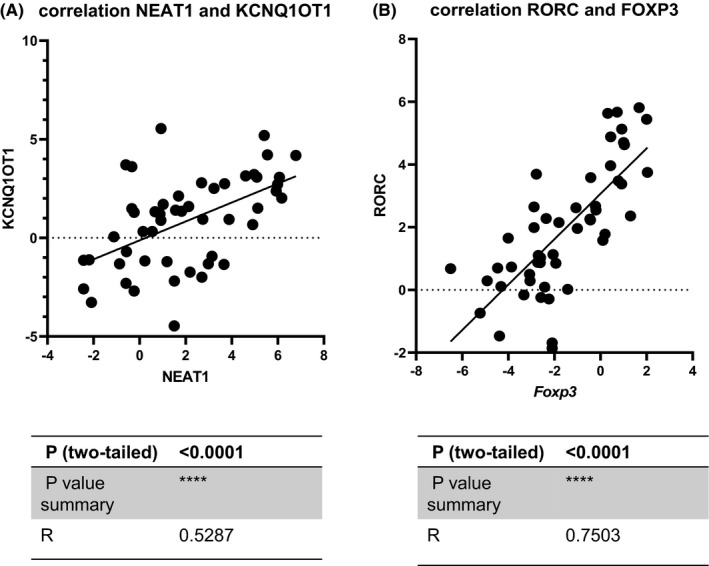
The scatter plots were drawn to demonstrate the positive correlation between lncRNAs NEAT1 and KCNQ1OT1 and the FOXP3 and RORC genes. A, The positive correlation of lncRNAs NEAT1 and KCNQ1OT1 (*R* = 0.5287, *p* < 0.0001). B, The positive correlation of FOXP3 and RORC (*R* = 0.7503, *p* < 0.0001) in case and control

RORC, an important transcription factor for Th17 cell development, whose overexpression can lead to demyelination of CNS lesions in RR‐MS patients, is a potential therapeutic target to prevent disease progression. Since Treg cells can regulate the balance of Th17 cells, RRMS patients had low expression levels of FOXP3 (*p* < 0.0001) and higher levels of RORC (*p* < 0.0001). In addition, a significant positive correlation was found between the levels of FOXP3 and RORC in patients with MS (*p* < 0.0001, *R* = 0.75). (Figures 5 and 6).

### 3.7 Cytokines assay demonstrates altered expression of TGF‐β and IL‐17 cytokines as critical indicators of Th17/Treg dysregulation in RR‐MS

**FIGURE 7:**
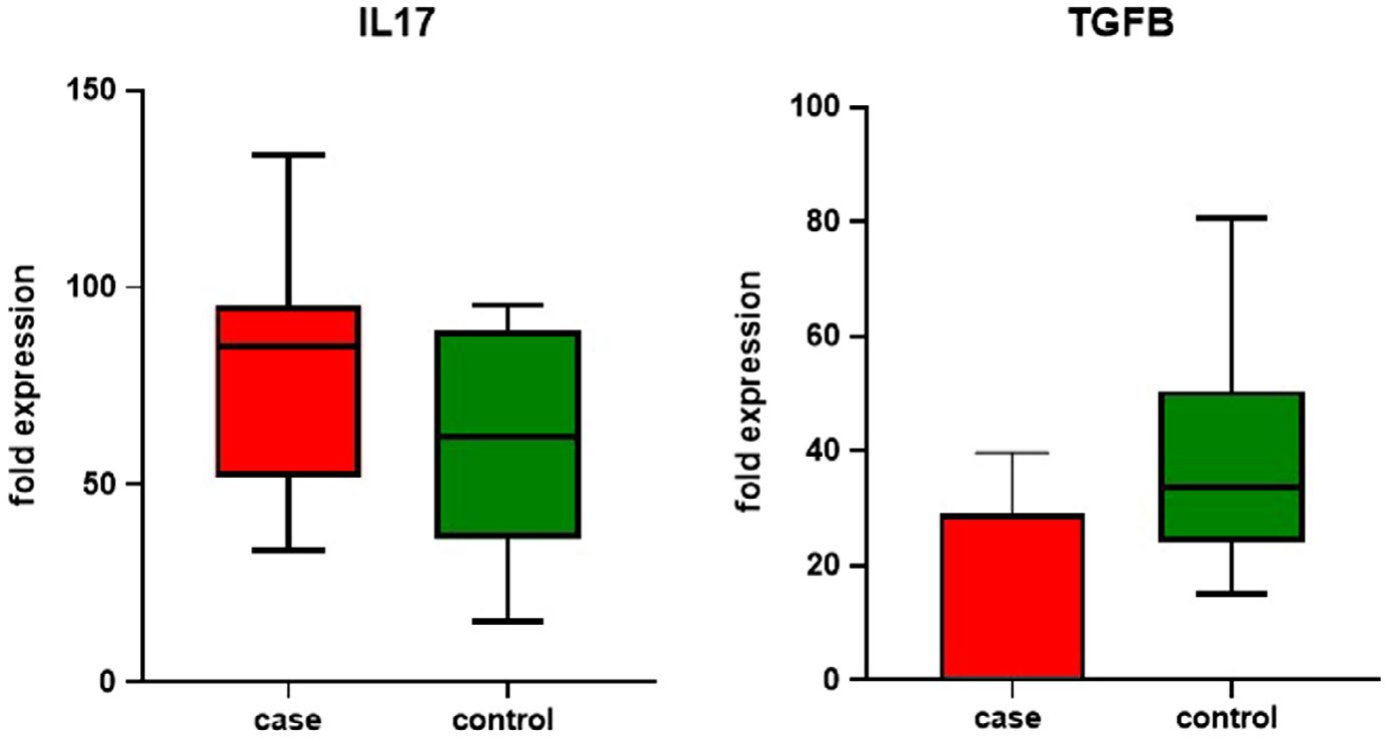
TGF‐β and IL‐17 Cytokine profiles assay as an instance of Th17/Treg imbalance in MS. TGF‐β and interleukin‐17 (IL‐17) were measured in a plasma sample of MS patients and healthy control by the ELISA method. The result exhibited that IL‐17 upregulation (*p* = 0.03) and downregulation of TGF‐β (*p* = 0.03) in RR‐MS compare to control

TGF‐β and interleukin‐17 (IL‐17) were measured in a plasma sample from MS patients and healthy controls using the ELISA method. The result showed that IL‐17 had an upregulation (*p* = 0.03) and downregulation of TGF‐β (*p* = 0.03) in RR‐MS compared to controls. These observations represent an imbalance in Th17/Treg cells. (Figure 7).

### 3.8 ROC analysis for lncRNAs and genes as clarified putative biomarker characterization in RR‐MS patients

**FIGURE 8:**
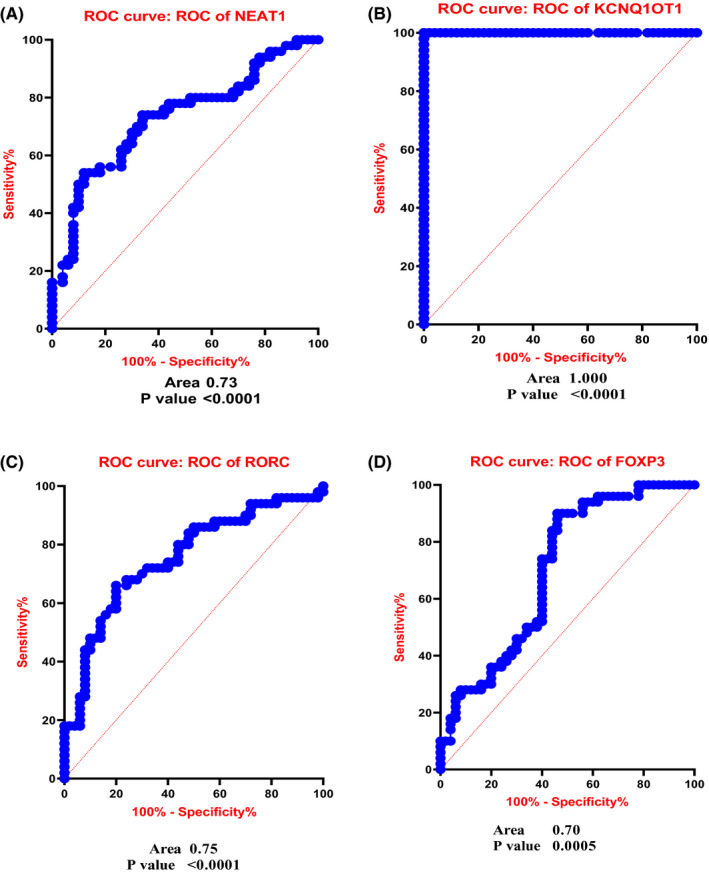
ROC curve analysis assessing indicated that these genes have relatively suitable sensitivity and specificity in MS diagnosis. The AUC for lncNEAT1 was 0.72.9 (A), KCNQ1OT1 was 1 (B), RORC was 0.75 (C) and FOXP3 was 0.70 (D)

The diagnostic value of NEAT1 for RR‐MS was assessed using a ROC curve. The AUC was 0.72.9 (95% CI 0.6293 to 0.8287, *p* < 0.0001), and the diagnostic value for KCNQ1OT1 was 1 (95% CI 1.000 to 1.000, *p* < 0.0001) indicating its suitability as a biomarker. This analysis was also performed for RORC with an AUC of 0.75 (95% CI 0.6598 to 0.8514, *p* < 0.0001), and the AUC for FOXP3 showed 0.70 (95% CI 0.5974 to 0.8046 and *p* = 0.0005) (Figure 8).

## 4 DISCUSSION

MS is a neurodegenerative and autoimmune disorder that results in damage to the central nervous system. The disease progresses rapidly without drug treatment. However, no conclusive cause for the development of MS has yet been found, and no early biomarkers for the disease have been discovered.^19^ Recently, it was discovered that lncRNAs control immune responses and inflammation in MS, suggesting that they may be the missing piece in the puzzle of MS etiopathology.^20^ Therefore, many efforts have been made in recent years to discover the MS molecular mechanism and function of ncRNAs underlying disease progression.^21^ Extensive data analysis has clarified many coding and non‐coding loci associated with MS demyelination, neuroinflammatory lesions and exocytopenia. Increased levels of Th17 cytokines (IL‐17A, IL‐17F and IL‐22) and decreased levels of Treg cytokines (IL‐10 and TGFB) were described to play a role in MS pathogenesis, including immune cell recruitment and exacerbation of CNS inflammation. In addition, the IL‐17 ratio was significantly increased in MS patients, implying that there is an imbalance between Th17 cells and Treg cells.^22^ Despite studies on the protein and signaling networks involved in Th17 subcellular differentiation, there is very little information on the role of lncRNA in controlling Th17 differentiation.^23^ Qiu et al^24^ reported that the lncRNA MEG3 functions as ceRNA and attenuates miR‐17 to regulate the Th17/Treg balance in asthma pathogenesis.

Using the GEO and lncRNA disease databases, we discovered several long non‐coding RNAs simultaneously involved in Th17 cell differentiation and autoimmune diseases, followed by a list of microRNAs in autoimmune diseases. We selected NEAT1 and KCNQ1OT1 as the lncRNAs with the most interactions with microRNAs in autoimmune diseases and the Th17 differentiation pathway. RNA‐seq analysis has also revealed that NEAT1 and KCNQ1OT1 are more upregulated in effector Th17 cells than in primary Th17 cells. Therefore, the most valuable new biomarkers for MS diagnosis would be those measured in PBMCs and serum. FOXP3, which reduces neuroinflammation and regulates the infection process, is responsible for the stability of Treg cells. In addition, data suggest that FOXP3 cells in peripheral blood contribute to MS tolerance.^25^ In 2019, Wang et al^26^ found that overexpression of the lncRNA DQ786243 in functional FOXP3+ Treg cells provides a novel benefit for oral lichen planus (OLP), including autoimmune and inflammatory diseases. Reduced FOXP3 expression decreases the differentiation of Treg cells, disrupting immune cell balance and homeostasis and increasing neuroinflammation.^27^ In our study, a decrease in FOXP3 was observed in patients with RR‐MS compared to healthy controls, which is consistent with other studies.^28^, ^29^ This leads to a loss of homeostasis between immune cells and the occurrence of inflammation in the nervous system.^27^ Our study also shows that there is a significant association between reduced FOXP3 gene expression and a positive family history of autoimmune disease (*p* = 0.03). In 2016, a study of different variants of FOXP3 found a significant difference in the distribution of the rs3761548 and rs2232365 alleles in MS patients compared to controls, suggesting that this polymorphism leads to suppression of FOXP3.^30^ The study found that 24% of MS patients who had recently been exposed to a viral disease such as COVID‐19 or influenza had a greater decrease in FOXP3 expression, which may be due to a significant reduction in the number of Treg cells following chronic neuroinflammation in the setting of viral infection.^31^ During these viral infections, the balance between Th17 and Treg cells is disturbed, predisposing an individual to the occurrence of MS. Several in vitro studies have shown that numerous HDAC or DNMT inhibitors, and unique immunomodulators, can neutralize proinflammatory cytokines (anti‐IL2 and anti‐TNF‐a) and restore FOXP3 expression and suppressive activity of Treg cells, suggesting that they may be a useful strategy for the treatment of autoimmune diseases.^32^

RORC is the major transcription factor for Th17 cells, and its transcription leads to the differentiation of CD^4+^ cells into them. Many studies have shown that the expression of RORC is increased in autoimmune diseases such as MS, RA, psoriasis and Crohn's disease.^33^ Studies show that in patients with MS, the population of subcellular Th17 cells and consequently the expression level of the IL17 gene are increased in PBMC, cerebrospinal fluid (CSF) and brain lesions or MS plaques, especially in the relapse phase compared to the recovery phase.^34^, ^35^ Therefore, considering the pathogenic and important role of subcellular Th17 cells in most autoimmune diseases, these cells appear to be a suitable therapeutic target for ameliorating or modifying autoimmune diseases. Accordingly, many studies have been conducted to discover Th17 signaling pathways in subcellular differentiation and to identify positive and negative regulators of Th17 differentiation.^36^ Since increased expression of the transcription factor, RORC leads to the differentiation of virgin CD^4+^ cells into Th17 cells, and Th17 cells are the most important cell at the beginning of the inflammatory process in the blood‐brain barrier, and increased expression of RORC is a primary factor in the pathogenesis of MS.^37^ The study also found that people with RR‐MS, who had a positive family history of autoimmune disease, had significantly increased RORC expression compared to people who did not have a positive family history of autoimmune disease. This can be demonstrated by the fact that these individuals have polymorphisms in their RORC gene that lead to increased expression of this gene.^38^

NEAT1 is a lncRNA that is abundant in the nucleus and has also been observed in the cytoplasm of mammalian cells. In general, each nucleus contains several paraspeckles, which are important for promoting transcription by RNA polymerase II. The formation and maintenance of paraspeckle structures require the presence of NEAT1.^39^ Paraspeckles serve as molecular centres for cellular processes that are often affected by neurodegeneration.^40^ NEAT1 levels in the CNS are altered in major neurodegenerative diseases and in certain human disease models. Dysregulation of NEAT1 expression is associated with neuronal damage; our knowledge of the role of NEAT1 in neurodegenerative diseases is still limited.^40^ Christiane Klec et al. reported that NEAT1 expression is increased in neurodegenerative diseases such as MS, Huntington, Parkinson's, Alzheimer's disease and amyotrophic lateral sclerosis.^41^ NEAT1 is also involved in the regulation of innate immune responses and non‐specific defence mechanisms in MS, and this lncRNA acts as a positive regulator of the inflammatory response.^42^ We used primers targeting the 5′ region of NEAT1 in this analysis, allowing us to detect both NEAT1 isoforms. According to the research by Zhang et al. NEAT1 expression increases in response to viral infections such as HIV‐1, influenza virus and herpes simplex virus, which upregulate antiviral genes such as interleukin 8. In these subjects, IL‐8 levels were significantly higher than in those who were not diagnosed with a neurological or autoimmune disease. Consequently, once the inflammatory process has begun, NEAT1 could accelerate the pathogenesis of MS by influencing macrophages and attracting them to the site of inflammation.^43^ The upregulation of NEAT1 was positively correlated with Th1‐associated TNF‐a and Th17‐associated IL‐17 to increase susceptibility to MS.^44^ Shui et al.^45^ reported increased expression of NEAT1 in RA and showed that suppression of NEAT1‐reduced Th17 differentiation. Upregulation of expression was also observed in our study, which is consistent with other studies.^41^ Dastmalchi et al^46^ confirmed overexpression of NEAT1 in PBMC from Iranian MS patients. Increased expression of NEAT1 was associated with enhanced immune signaling and expression of cytokines and chemokines, including IL‐6 and CXCL10, while IL‐1 and TNF‐a were suppressed.^47^ The expression level of NEAT1 in female patients was found to be higher than in affected male patients, suggesting that NEAT1 acts differently in the female immune system than in the male. The increase in NEAT1 expression suggests a coincidence with the onset of RR‐MS and greater disease severity in women.^46^ In our study, a significant association was found between NEAT1 expression in families with a positive history of the disease, such that NEAT1 expression was increased in individuals with a family background of autoimmune disease. Furthermore, based on the results, this study found that NEAT1 could be effective in the possible diagnosis of the disease with 72% specificity and sensitivity. Increased NEAT1 expression likely leads to suppression of Treg cells through H3K4me3 methylation in FOXP3.^48^ According to a report on inflammatory bowel disease, increased NEAT1 expression is involved in inflammatory responses by controlling exosome‐mediated macrophage polarity.^49^

KCNQ1 opposite strand/antisense transcript 1 (KCNQ1OT1) has a function in epigenetic processes and represses gene expression. KCNQ1OT1 controls the transcription of many genes in chromosomal domains by interacting with chromatin. The number of fibroblasts has been linked to the number of KCNQ1OT1, which served as an indicator of airway remodelling. KCNQ1OT1 is a 91 kb transcript that is antisense to the intron of 10 KCNQ1 genes.^50^ As epigenetic regulators, (KCNQ1OT1) plays a key role in gene regulation by binding to the repressive polycomb complex 2 (PRC2) and methyltransferase G9a (EHMT2).^9^ Research has suggested that the key function of KCNQ1OT1 in long QT syndrome, cataracts, cancers and Beckwith‐Wiedemann also some observations have suggested the KCNQ1OT1 as an oncogenic molecule in cancer.^51^, ^52^, ^53^, ^54^ There is a significant difference in KCNQ1OT1 expression in serum samples from asthmatic children with airway remodelling compared with those without airway remodelling.^54^ Singh et al^55^ in 2015 reported that KCNQ10T1 and NEAT1 were present in CD^4+^ and CD^8+^ cells. Increased expression of NEAT1 and KCNQ10T1 is observed in the lncRNA expression profile of peripheral blood from MS patients. KCNQ1OT1, which is highly expressed in TBI mice, and its suppression could reduce neurological disorders, inflammation, and the suppression of cerebral oedema through microglia activity reduction.^56^ miR‐200 plays a neuroprotective role in stroke and is repressed by the upregulation of KCNQ1OT1. Studies show that KCNQ1OT1 is significantly increased in type 2 diabetes mellitus (T2D).^57^ Increased expression of KCNQ1OT1 in people with RR‐MS was observed in our study. In this study, increased expression of KCNQ1OT1 in MS was investigated, and it is suggested that it plays role in the pathogenesis of MS through the effect on Th17/Treg imbalance. The ROC curve for KCNQ1OT1 indicates 100% specificity and sensitivity, which may indicate the significant function of this lncRNA in disease development and diagnosis. Correlation analysis of gene expression revealed that NEAT1 and KCNQ1OT1 lncRNAs display a significant and positive relationship with each other, indicating the cooperation of these two lncRNAs in MS pathogenesis.

## 5 CONCLUSIONS

These results suggest for the first time that the lncRNA NEAT1 and the lncRNA KCNQ1OT1 influence MS pathogenesis through an imbalance between Th17 and Treg and can be considered a potential anti‐inflammatory target. Considering the central and pathogenic role of Th17 cell subtype in autoimmune diseases, lncRNAs are also suitable as a therapeutic agent. There are few studies on the lncRNAs network involved in the differentiation of Th17 cell lines. Therefore, in the future, these data can be used to predict and develop effective drugs that inhibit the differentiation of pathogenic Th17 subtypes in autoimmune diseases, thereby alleviating or modifying these diseases. Finally, this study is the first to simultaneously investigate the expression profile of this gene set on MS as a molecular mechanism involved in the imbalance between Th17 and Treg. It suggests that KCNQ1OT1 and NEAT1 play an essential role in the development of the intercellular imbalance between Th17 and Treg and that these lncRNAs can be used for pharmacological, therapeutic and diagnostic purposes.

## CONFLICT OF INTEREST

The authors declare that they have no conflict of interest.

## AUTHOR CONTRIBUTIONS

Elham Karimi: Conceptualization (Lead), Methodology (Lead), Resources (Lead), and Writing – original draft (Lead). Hanieh Azari: Investigation (Lead) and Writing – original draft (Lead). Aminreza Nikpoor and Ahmad Tahmasebi^:^ Formal analysis (Equal) and Software (Equal). Ahmad agha Negahi: Resources (Equal). Nima Sanadgol: Writing – review & editing (Lead). Mohammad Shekari: Project administration (Equal) and Supervision (Equal) and Pegah Mousavi: Funding acquisition (Lead), Project administration (Equal), Supervision (Equal) and Writing – review & editing (Lead).

## Data Availability

The data that support the findings of this study are available from the corresponding author upon reasonable request.
